# Frontiers and hotspots of ^18^F-FDG PET/CT radiomics: A bibliometric analysis of the published literature

**DOI:** 10.3389/fonc.2022.965773

**Published:** 2022-09-13

**Authors:** Xinghai Liu, Xianwen Hu, Xiao Yu, Pujiao Li, Cheng Gu, Guosheng Liu, Yan Wu, Dandan Li, Pan Wang, Jiong Cai

**Affiliations:** ^1^ Department of Nuclear Medicine, Affiliated Hospital of Zunyi Medical University, Zunyi, China; ^2^ The First Clinical College, Zunyi Medical University, Zunyi, China; ^3^ Department of Obstetrics, Zunyi Hospital of Traditional Chinese Medicine, Zunyi, China

**Keywords:** bibliometric analysis, ^18^F-FDG, PET/CT, radiomics, hot topics

## Abstract

**Objective:**

To illustrate the knowledge hotspots and cutting-edge research trends of ^18^F-FDG PET/CT radiomics, the knowledge structure of was systematically explored and the visualization map was analyzed.

**Methods:**

Studies related to ^18^F-FDG PET/CT radiomics from 2013 to 2021 were identified and selected from the Web of Science Core Collection (WoSCC) using retrieval formula based on an interview. Bibliometric methods are mainly performed by CiteSpace 5.8.R3, which we use to build knowledge structures including publications, collaborative and co-cited studies, burst analysis, and so on. The performance and relevance of countries, institutions, authors, and journals were measured by knowledge maps. The research foci were analyzed through research of keywords, as well as literature co-citation analysis. Predicting trends of ^18^F-FDG PET/CT radiomics in this field utilizes a citation burst detection method.

**Results:**

Through a systematic literature search, 457 articles, which were mainly published in the United States (120 articles) and China (83 articles), were finally included in this study for analysis. Memorial Sloan-Kettering Cancer Center and Southern Medical University are the most productive institutions, both with a frequency of 17. ^18^F-FDG PET/CT radiomics–related literature was frequently published with high citation in *European Journal of Nuclear Medicine and Molecular Imaging* (IF9.236, 2020), *Frontiers in Oncology* (IF6.244, 2020), and *Cancers* (IF6.639, 2020). Further cluster profile of keywords and literature revealed that the research hotspots were primarily concentrated in the fields of image, textural feature, and positron emission tomography, and the hot research disease is a malignant tumor. Document co-citation analysis suggested that many scholars have a co-citation relationship in studies related to imaging biomarkers, texture analysis, and immunotherapy simultaneously. Burst detection suggests that adenocarcinoma studies are frontiers in ^18^F-FDG PET/CT radiomics, and the landmark literature put emphasis on the reproducibility of ^18^F-FDG PET/CT radiomics features.

**Conclusion:**

First, this bibliometric study provides a new perspective on ^18^F-FDG PET/CT radiomics research, especially for clinicians and researchers providing scientific quantitative analysis to measure the performance and correlation of countries, institutions, authors, and journals. Above all, there will be a continuing growth in the number of publications and citations in the field of ^18^F-FDG PET/CT. Second, the international research frontiers lie in applying ^18^F-FDG PET/CT radiomics to oncology research. Furthermore, new insights for researchers in future studies will be adenocarcinoma-related analyses. Moreover, our findings also offer suggestions for scholars to give attention to maintaining the reproducibility of ^18^F-FDG PET/CT radiomics features.

## Introduction

Fluorine-18 fluorodeoxyglucose positron emission computed tomography (^18^F-FDG PET/CT) is one of the most widely used metabolic imaging methods in nuclear medicine, which can reflect the heterogeneity of glucose metabolism between tumor cells and normal cells in an early and quantitative manner (1). Generally, this technique is widely used to evaluate the differential diagnosis of benign and malignant tumors, the staging and restaging of malignant tumors, the evaluation of treatment efficacy, prognosis prediction, and so on (2, 3). Radiomics is a relatively broad concept, and its application includes traditional imaging techniques, such as computed tomography (CT), magnetic resonance imaging (MRI), and ultrasound. As the frontier of molecular imaging, nuclear medicine, represented by single-photon emission CT (SPECT) and PET/CT, has significant advantages in a series of clinical problems including efficacy evaluation of refractory malignant tumors, differential diagnosis of heterogeneous cells, and disease prognosis and survival evaluation (4, 5). Recent studies have demonstrated the advantages or superior performance of radiomics over traditional manual readings (6–8). As far as manual reading is concerned, the accuracy and reliability of the image report largely depend on the clinical experience and expertise of the doctor. Doctors who are inexperienced and unskilled in using image analysis tools often produce low-quality inspection reports, which may lead to misdiagnosis or even missed diagnosis. That might be the reason why the result of PET/CT diagnosis varies from doctor to doctor. For senior doctors, it is a basic literacy to accurately describe the examination results according to the visible imaging changes at the lesions. Unfortunately, human eyes cannot distinguish the subtle changes specific to each pixel in an image, whereas this can be done entirely through formal machine learning and machine recognition. In previous clinical studies, CT and MRI radiomics analysis has shown high accuracy in distinguishing benign and malignant tumors and in evaluating the efficacy and prognosis after treatment (9–12). In recent years, radiomics has also been used to assist in the diagnosis of endoscopic ultrasound images and to identify whether it is a COVID-19 infection by chest x-ray (13, 14). Up to now, radiomics-related literature has been analyzed by knowledge visualization maps from a macroscopic perspective. Nonetheless, bibliometric methods have not yet been used to summarize the literature on specific metabolic imaging technology.

PET/CT imaging can clearly and intuitively reflect the metabolic changes of tumor cells and detailed metabolic image changes in the establishment of treatment plans for malignant tumors, identification of benign and malignant pulmonary nodules (15), identification of radiation necrosis and tumor recurrence in glioma patients (16), non-invasive prediction of epidermal growth factor receptor (EGFR) mutations in lung adenocarcinoma (17), and other clinical problems; the diagnostic accuracy of PET/CT radiomics analysis is expected to be superior to traditional methods. As the process of precision medicine deepens, scholars have been interested in analyzing ^18^F-FDG PET/CT metabolic images according to the core technologies of radiomics to assist in clinical diagnosis, the establishment of treatment decisions and prediction of prognosis, etc. and have achieved promising results with the number of related research publications increasing year by year (18–22).

Bibliometric is a science that uses quantitative methods such as mathematics and statistics to study the distribution laws and quantitative relationships of documents. In order to encourage researchers from various disciplines to actively and creatively participate in the practice of ^18^F-FDG PET/CT radiomics, this present study mainly uses the CiteSpace software to visualize and analyze the related research of ^18^F-FDG PET/CT radiomics, sorting out the current situation and future development trend of this field. The aim of this study is to offer clinicians an objective summary of the development processes and research hotspots and provide reference and scientific bases for scholars to refine the research direction.

## Methods

### Data retrieval strategy

Before searching the literature, we contacted the Information Department of the Zunyi Medical University Library to interview professional workers about data search strategies. Based on the interview, we expanded the synonyms and subordinate terms based on the MeSH database (https://www.ncbi.nlm.nih.gov/mesh, USA), and, finally, we determined the search terms and the combined search form. The retrieval formula can be described as follows: TS=((^18^F-FDG OR FDG OR Fluorodeoxyglucose OR Fluorine 18 fluorodeoxyglucose OR 2 Fluoro 2 deoxy D glucose) AND (Positron Emission Computed Tomography OR PET OR PET/CT OR PET-CT) AND Radiomic*). The Web of Science (WOS) database was used to search the Science Citation Index Expanded (SCI-E) from its core collection (WoSCC) database, all data were searched by our corresponding authors independently (Prof. Pan Wang and Dr. Jiong Cai, Affiliated Hospital of Zunyi Medical University, Zunyi, China). Relevant data published between 2013 and 2021 were obtained. The process of data downloading and literature searching was all completed on 1/3/2022, in order to preclude potential bias caused by frequently updated data. The proofreading notices, editing materials, conference papers, and retraction notices were excluded, and, by reading the titles and abstracts, obviously irrelevant literatures were excluded as well. Original articles and reviews were included for utilization for the bibliometric analysis. Details of data searching were presented in **Supplementary Figure 1**, and general data from WoSCC were shown in **Supplementary Tables 1, 2**. In case that a disagreement occurs during the execution of the above steps, either the two corresponding authors discussed with each other to resolve problems or they consulted the third author to assist in judgment.

### Analysis method

**Figure 1:**
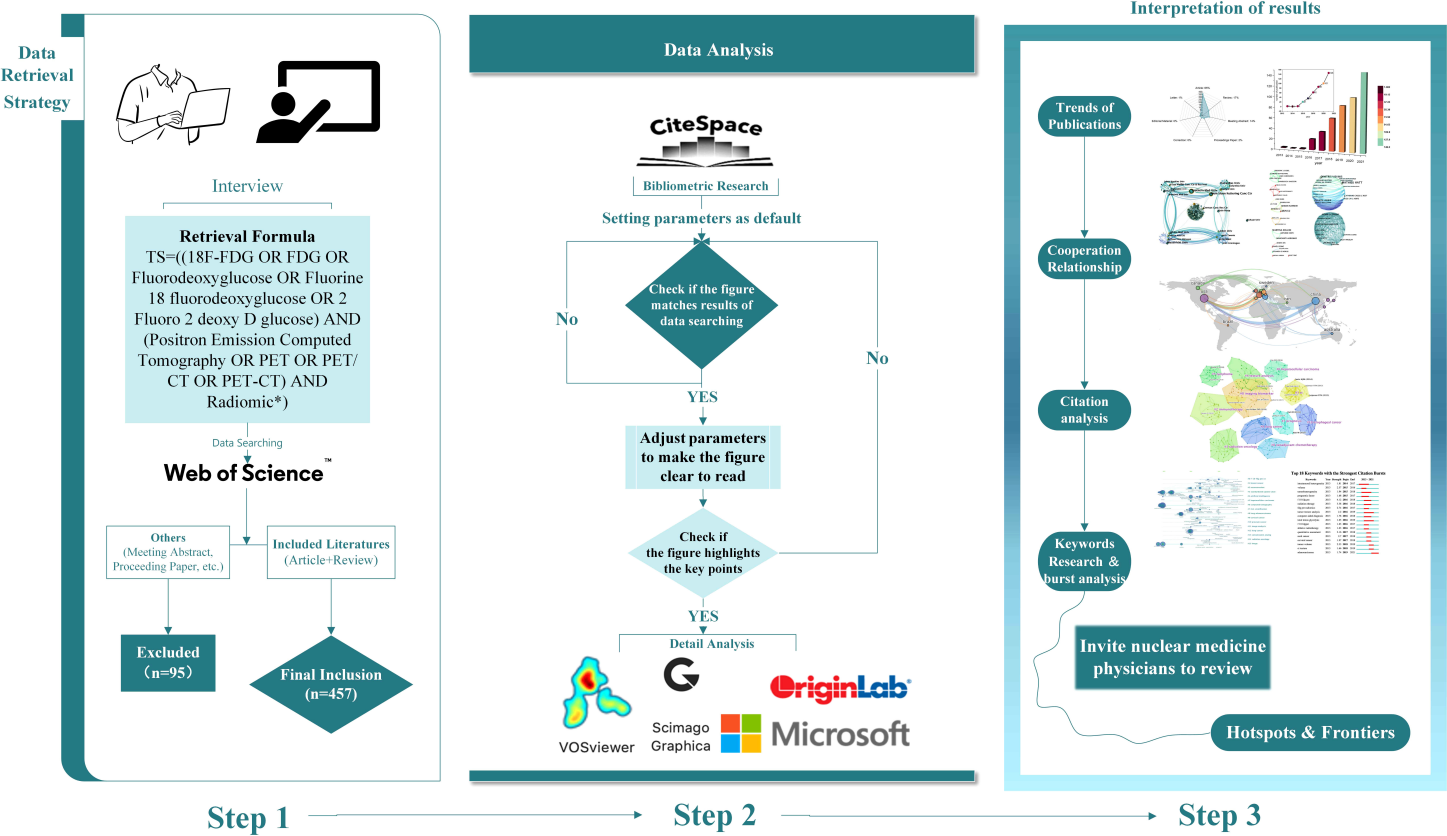
Flow chart of this bibliometric analysis. (Step 1) Data retrieval strategy of published literatures, showing the detailed process of screening and inclusion. (Step 2) Data analysis of this study, including bibliometric analysis and data processing. (Step 3) Interpretation of results, containing a brief view of results and the final process of drawing conclusions.

All WoSCC data were transformed into text containing information of authors, research institutions, subjects, years, keywords, abstracts, journals, volumes, and page numbers. We created a spider map and bar charts to identify publication volumes and annual trends by using OriginPro 2021 (OriginLab Corporation, USA). CiteSpace 5.8.R3 (Drexel University, USA) was applied to identify visualized map: 1) Analyzation map of the overall research status was generated by illustrating the distribution of disciplines, journals, countries/regions, institutions, and the contribution of authors; 2) keyword co-occurrence and clustering analysis were performed to anchor research hotspots; and 3) co-citation and citation burst analysis were performed to target frontiers in the field. VOSviewer 1.6.16 (Leiden University, Netherlands) was used to assist in constructing visualization maps of countries/regions and keyword co-occurrence. Scimago Graphica 1.0.18 (https://www.graphica.app/, USA) was applied to form a world map depicting the publication counts of each country. All software used in this study was performed on Windows 10 (64-bit) Chinese version operating system (Microsoft, USA). When importing the data into bibliometric analysis, we set the parameters as default to obtain original maps. We checked the original maps to see if the intellectual structure matched the results of data searching. After that, adjusted parameters to ensure the generated graphs were clear enough to highlight key points. After all analyses were finished, we invited three nuclear medicine physicians to review if the results are objective and fit the developing trends in this field based on their professional knowledge. The flow chart of this bibliometric analysis is shown in **Figure 1**.

## Results

### Temporal trends of publications

**Figure 2:**
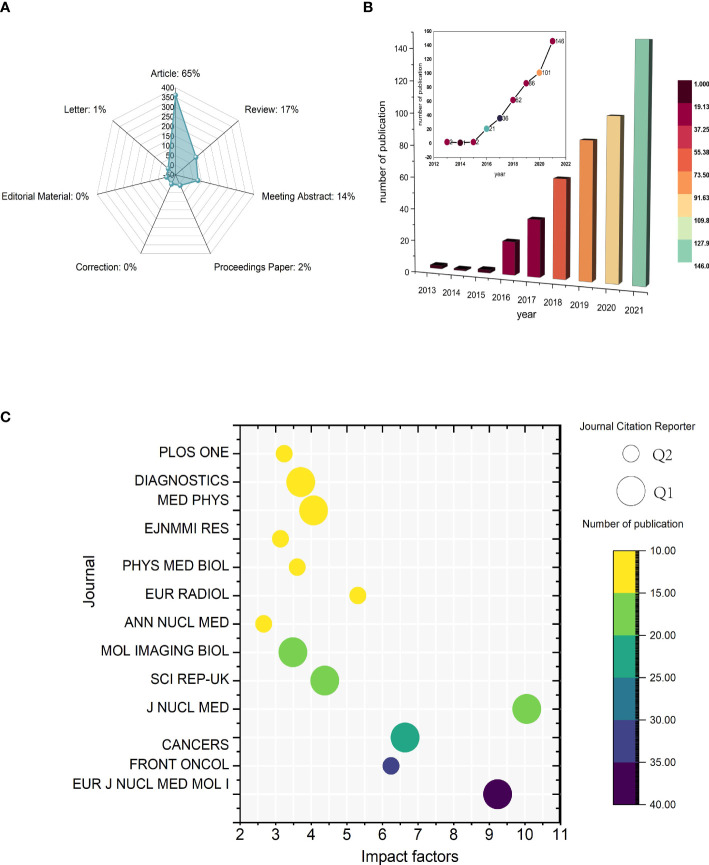
Temporal trends of publications of ^18^F-FDG PET/CT radiomics research. **(A)** Category of all published literatures. **(B)** Annual publications and temporal trends of ^18^F-FDG PET/CT radiomics research. **(C)** Bubble plot of journals with more than 10 publications. The size of bubbles represents classification from Journal Citation Reports and the colors are related to the number of publications.

A total of 552 publications were initially generated through the WoSCC search (**Figure 2A**). After excluding 95 publications, 457 publications were finally included in this study, comprising 361 original articles and 96 reviews. From 2013 to 2015, there were few relative studies, indicating low research interest in this area. From 2016 onward, the number of papers on ^18^F-FDG PET/CT radiomics began to increase rapidly. In 2021, the importance of ^18^F-FDG PET/CT radiomics research has been noticed, with 146 publications annually—an increase of more than 40% over the same period in the previous year. It is the first high-yield stage in this field, showing a significant trend of steady growth in the number of publications (**Figure 2B**).

### Distribution of disciplines and productive journals

All articles in this study were published in 134 journals, 13 of which were with more than 10 articles published **(** **Figure 2C** **)**. The impact factor (IF) and journal quartile were obtained from Journal Citation Reports 2020 (23). The top 3 prolific journals were *European Journal of Nuclear Medicine and Molecular Imaging* (IF9.236), *Frontiers in Oncology* (IF6.244), and *Cancers* (IF6.639). The top journal with the highest IF was *Journal of Nuclear Medicine* (IF10.057). In addition, journals with more than 10 publications were all classified in Q1 or Q2. Specifically, with 53.84% (7/13) being Q1 and 46.15% (6/13) being Q2 among these journals, the results indicate that the above journals have strong academic performance in ^18^F-FDG PET/CT radiomics research.

**Figure 3:**
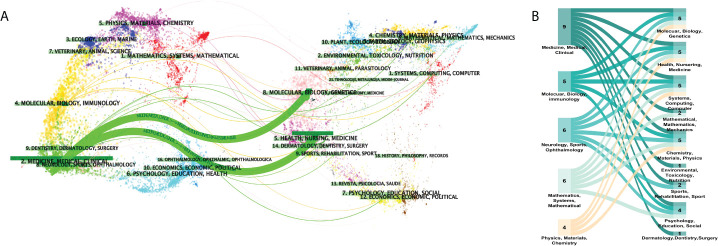
Distribution of disciplinaries. **(A)** Dual-visualization map. The left part was the targeted literature, whereas the right part was the source literature. Each dot represents one journal. On the left, there are the citing journals of this field and, on the right, lay the cited journals in this field. The waves linking to two sides mean that the publications on the journals on the left side may cite publications from the journals on the right-hand side. **(B)** Sankey diagram. The numbers on the graph show the amounts of publications in each disciplinary. Disciplinaries shown on the left are the subjects of citing literature and on the right-hand side lays the subjects of cited ones. The color represents citation relationships of corresponding disciplinaries.

Academic journals are an important mass media for the academic communication of disciplinary knowledge, and the journal distribution of literature reflects the information related to the research status to a certain extent. Scholars can gain a simple understanding of the current research status by considering the number of publications in different journals. In the dual-visualization map **(** **Figure 3A** **)**, there are two main links identified, with green color: Medicine/Medical/Clinical journals frequently cite studies published in Health/Nursing/Medicine journals and studies published in Medicine/Medical/Clinical journals are also cited in the studies published in Molecular/Biology/Genetics journals. More clear details can be seen in **Figure 3B**, showing the connection between disciplines.

### Cooperation between countries/regions and institutions

All publications were distributed among 45 countries (or regions) and 214 institutions. The nodes in the visualization map represent countries/regions/institutions, and the connection line represents the strength of the relationship between the two nodes. The larger the circle of the node, the greater the volume of published documents. The thicker the connection, the closer the cooperative relationship.

### Active countries

**Figure 4:**
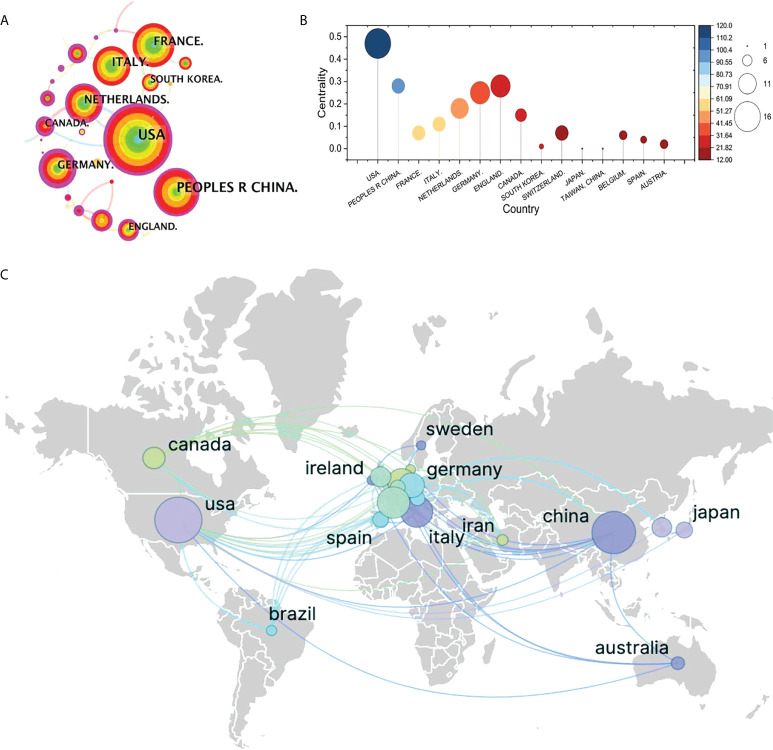
Visualization map of cooperation between countries. **(A)** Collaboration visualization map of countries of bibliometric app (CiteSpace), *N* = 641, *E* = 560 (*N* represents the number of visualization map nodes. *E* represents the number of connections). The nodes in the visualization map represent countries/regions/institutions, and the connection line represents the strength of the relationship between the two nodes. The larger the circle of the node, the greater the volume of published documents. The thicker the connection, the closer the cooperative relationship. **(B)** Top 10 most productive countries involved in ^18^F-FDG PET/CT radiomics research. **(C)** Collaborative research relationships between countries based on VOSvieser and Scimago Graphica.

The top contributor was the United States (120 publications, 26.26%), followed by China (100 publications, 21.88%), France (58 publications, 12.69%), Italy (56 publications, 12.25%), and Netherlands (43 publications, 9.40%) (**Figure 4A**). Although neither China (centrality = 0.28) nor Germany (centrality = 0.25) have the highest publication frequency, we still identify the three countries as critical nodes according to their high centrality (**Figure 4B**), which means that they play a significant role in the field of ^18^F-FDG PET/CT radiomics. The above results demonstrate that ^18^F-FDG PET/CT radiomics is increasingly receiving widespread attention from global scholars and that extensive research has been conducted recently, especially in the United States. Other high-quality publications were mainly completed by Chinese, French, German, and Dutch researchers. The cooperation between countries/regions across the world was shown in **Figure 4C**.

### Productive institutions

**Figure 5:**
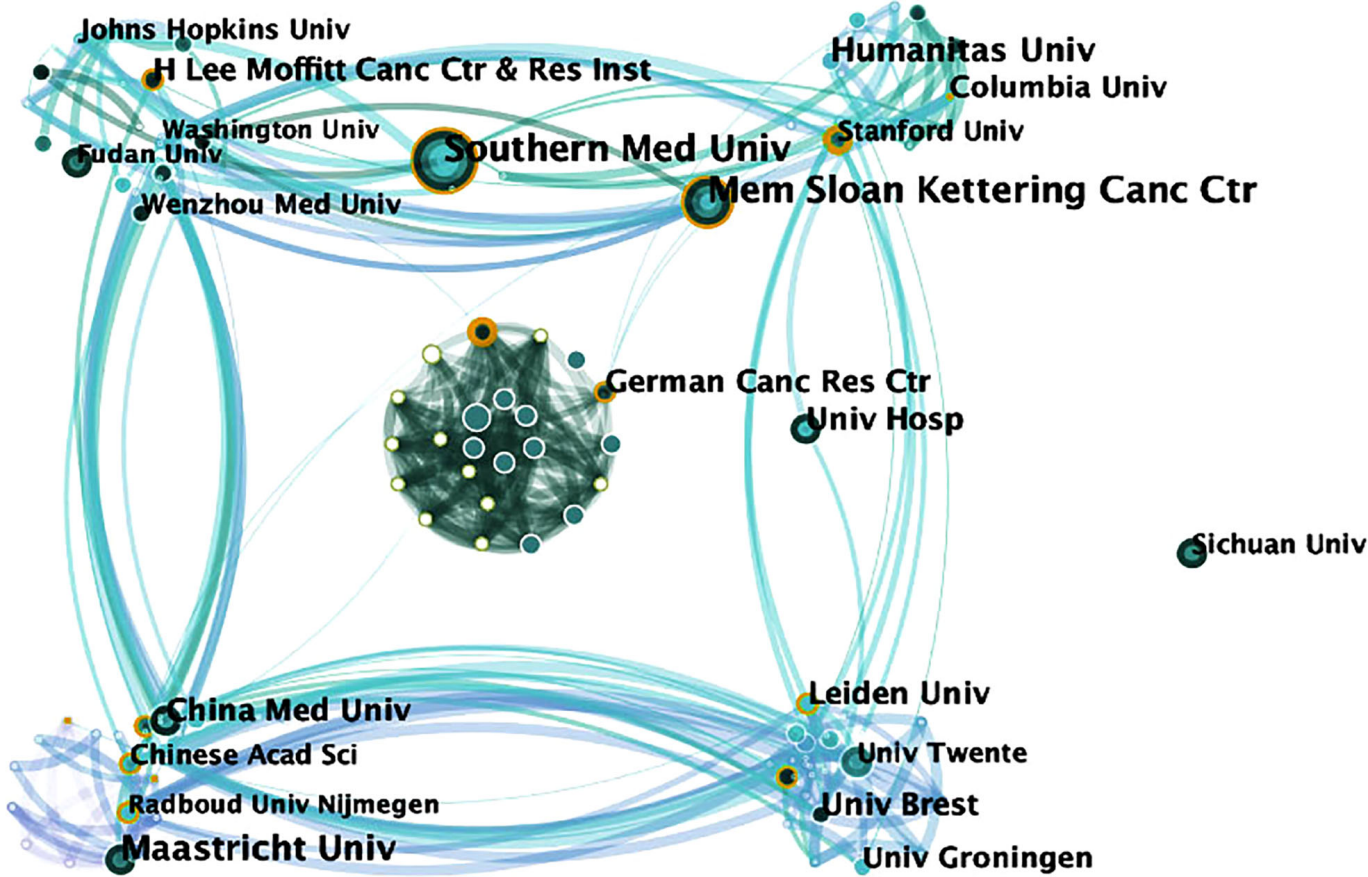
Map of active institutions from 2013 to 2021. The size of each circle is proportional to the article counts. The thickness of the curved connecting line represents the collaborative intensity between institutions.

The visualization map shows the 214 institutes which made contributions to ^18^F-FDG PET/CT radiomics research **(** **Figure 5** **).** Institutions with the highest frequency were shown in **Table 1**, and full names of the institutions are shown in **Supplementary Table 3**. The Memorial Sloan-Kettering Cancer Center and Southern Medical University are the most productive institutions with 17 publications, followed by Maastricht University (frequency = 14), H. Lee Moffitt Cancer Center and Research Institute (frequency = 13), and Humanitas University (frequency = 12). We investigated the possible impact of research published by institutions according to the centrality of the visualization map. The German Cancer Research Center (centrality = 0.13), the University of Groningen (centrality = 0.13), and the Memorial Sloan-Kettering Cancer Center (centrality = 0.11) tend to be the leading driving force and still dominate in this research field among top institutions.

**Table 1:** Institutions with the highest frequency related to ^18^F-FDG PET/CT radiomics.

Rank	Institution	Country	Frequency	Degree	Centrality
1	Memorial Sloan-Kettering Cancer Center	USA	17	11	0.11
2	University of Groningen	Netherlands	11	12	0.13
3	Leiden University	Netherlands	11	12	0.19
4	German Cancer Research Center	Germany	11	17	0.13
5	Stanford University	USA	9	9	0.19
6	Chinese Academy of Sciences	China	7	8	0.15
7	Helmholtz-Zentrum Dresden Rossendorf	Germany	7	21	0.21
8	Vrije University Amsterdam Medical Center	Germany	5	14	0.13
9	University of Michigan	USA	2	12	0.16

### Contribution of authors

**Figure 6:**
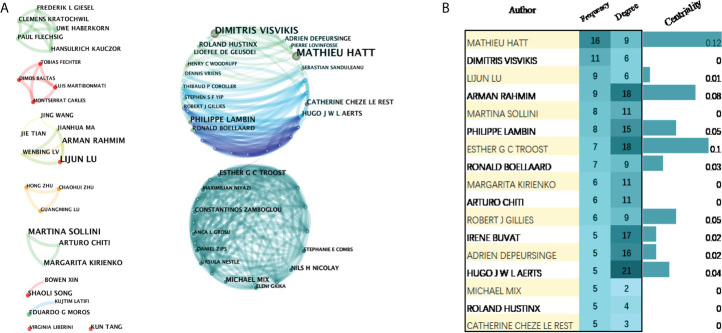
Visualization map of active authors in the field of ^18^F-FDG PET/CT radiomics. **(A)** Cooperation of authors. **(B)** Authors with more than five publications.

Focusing on the research contributions of authors helps us to quickly classify scholars who are active in the discipline at this stage. A total of 288 authors have made contributions to ^18^F-FDG PET/CT radiomics research. The top 10 prolific authors and top 10 cited authors were listed in **Supplementary Tables 4, 5**. Mathieu Hatt, the most productive author from Australia, published 16 articles, followed by Dimitris Visvikis (11 articles, France), Lijun Lu (9 articles, China), Arman Rahmim (9 articles, USA), and Martina Sollini (8 articles, Italy) (**Figure 6A**). Among the authors with a relatively larger number of publications, Mathieu Hatt and Esther G. C. Troost have greater influence in the field, with the centrality ≥0.1 in the visualized map of our bibliometric analysis (**Figure 6B**).

### Research of keyword

Keywords are a high-level summary and condensation of the topic in an article. Those with high frequency in different periods reflect the core issues in the field, whereas their clustering view highlights key nodes and important connections, revealing the research theme of a knowledge field and its evolution process.

### Keyword co-occurrence

The visualized/visualization map constructed based on the literature includes 298 nodes, and the nodes are closely related to form a complex relationship network, indicating that the literature has a wide range of research **(** **Supplementary Figure 2A** **)**. According to the density map, keyword nodes represented by “radiomics”, “CT”, “images”, “diagnosis”, and “features” are closer to red color **(** **Supplementary Figure 2B** **)**, suggesting that they are the frequently occurring keywords of ^18^F-FDG PET/CT radiomics.

### Keyword clusters

**Figure 7:**
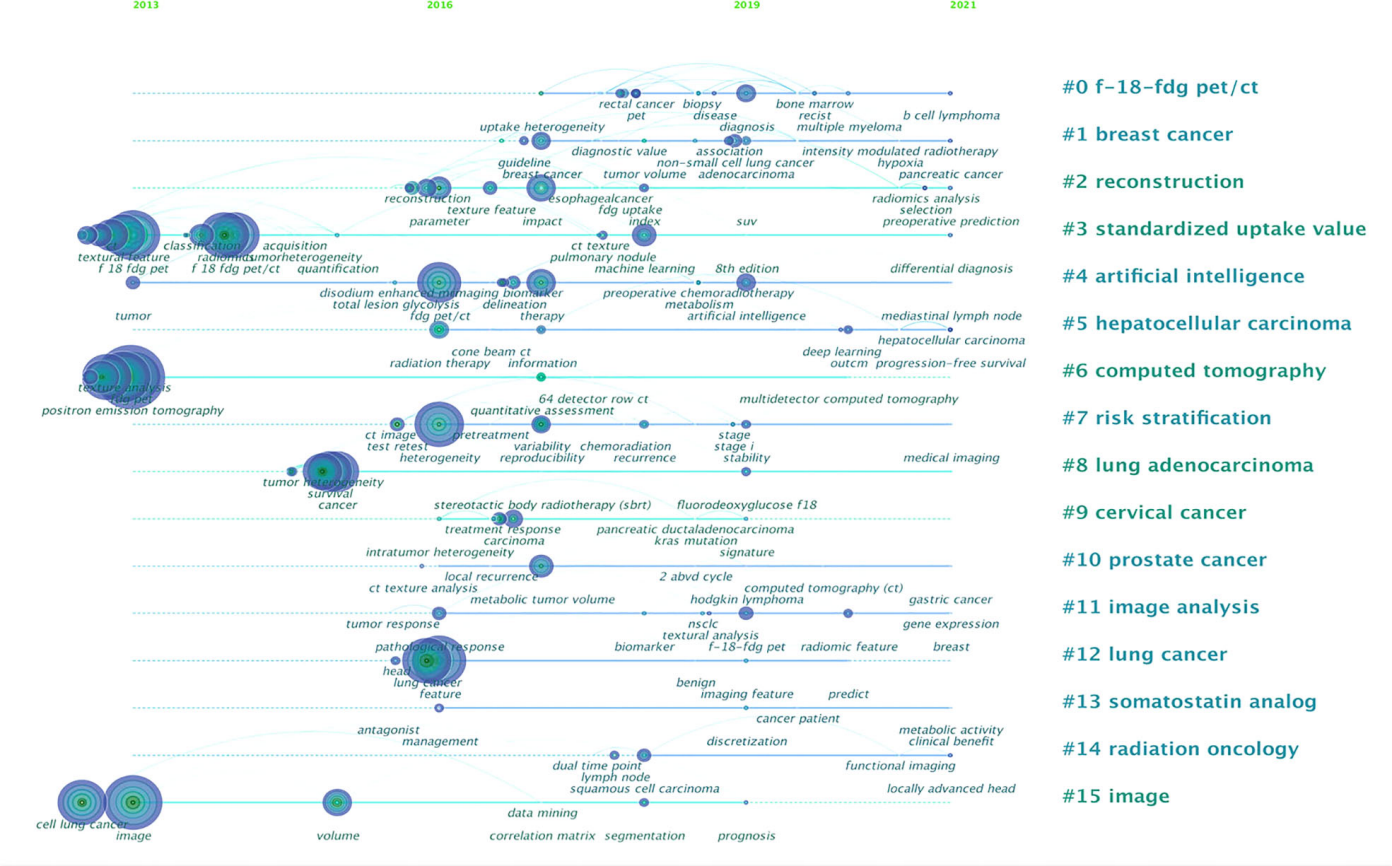
Analysis of all keywords in studies related to ^18^F-FDG PET/CT radiomics research. In the timeline view of keyword cluster analysis, there are *N* = 298 and *E* = 598.

The high-frequency keywords and their clusters at different times are shown in **Figure 7**. Analysis of keywords found that the content of the literature mostly focused on the research methodology of radiomics (keywords: positron emission tomography, FDG PET, F 18 FDG PET/CT, computed tomography, etc.), and scholars were very concerned about the core analysis content of PET/CT radiomics (keywords: texture analysis and textural feature). Data related to diagnosis and prognosis are valued in terms of evaluation indicators (keywords: heterogeneity, survival, and prognostic value). On the basis of the co-occurrence network, the log-likelihood ratio (LLR) algorithm was used to cluster the keywords. In the timeline view clusters, the Silhouette (S) value refers to the average contour value of the cluster. It is generally considered that, with an S > 0.5, the cluster is reasonable, whereas with an S > 0.7, the cluster is convincing. In our study, S = 0.9316, indicating that the clustering is efficient and convincing. Around 2013, scholars had just started to contact ^18^F-FDG PET/CT radiomics research, and they were typically interested in “standardized uptake value”, “computed tomography”, and “image”; by 2016, the keywords involved in the research had changed and were mainly clustered as “reconstruction”, “artificial intelligence”, “risk stratification”, “lung adenocarcinoma”, and “lung cancer”. We can see that lung cancer is the first single disease to be cited in ^18^F-FDG PET/CT radiomics field; since then, ^18^F-FDG PET/CT radiomics research topics have become increasingly broad and have begun to focus on different directions, such as “hepatocellular carcinoma”, “cervical cancer”, and “prostate cancer”. The actual frequency numbers of each term of clusters can be seen in **Supplementary Table 6**.

### Literature co-citation analysis

**Figure 8:**
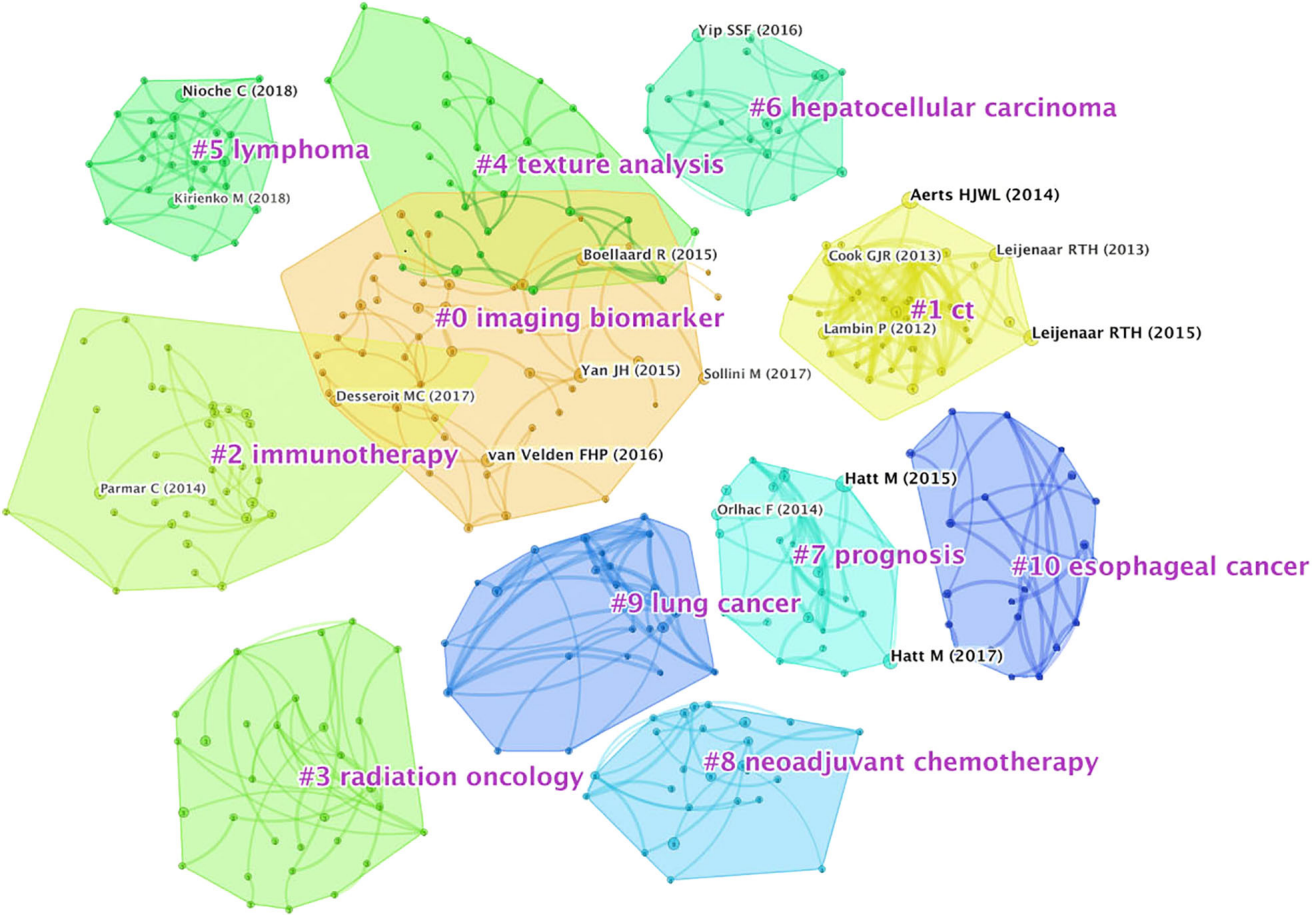
Analysis of author co-citation related to ^18^F-FDG PET/CT radiomics research. *N* = 482, *E* = 837. The connection between the nodes represents the closeness in the relationship. The tighter the relationship is, the thicker the line is. The blue “#” labels show the cluster name, and the blocks of the same color are divided into studies on the same topic.

Normally, looking into the co-citation relationship allows us to explore the development and evolutionary dynamics of a particular study (24). Each node in the visualization map represents a paper and the first author’s information was listed briefly. The connection between the nodes represents the closeness in the relationship. The tighter the relationship is, the thicker the line is. The blue “#” labels show the cluster name, and the blocks of the same color are divided into studies on the same topic. Most of the studies were divided into 11 core clusters, and to be specific, radiation oncology (#3) is the subject most related to ^18^F-FDG PET/CT radiomics research, indicating that the broad application of artificial intelligence analysis based on machine learning plays a significant role in the evaluation of tumor radiation therapy efficacy, target delineation, prognosis evaluation, etc. CT (#1) and texture analysis (#4) are the basic method or technology of medical examination required for carrying out ^18^F-FDG PET/CT radiomics research. Imaging biomarkers (#0) are core evaluation methods of radiomics. Hepatocellular carcinoma (#6), lymphoma (#5), lung cancer (#9), and esophageal cancer (#10) are the most researched diseases at this stage. Neoadjuvant chemotherapy (#8) and prognosis (#7) indicate that scholars pay close attention to the application of ^18^F-FDG PET/CT radiomics on efficacy assessment and prognostic analysis **(** **Figure 8** **)**. Generally speaking, in a cluster analysis, 0 ≤ Q ≤ 1 and Q > 0.3 indicate that there are significant differences in the divided clusters. In this study, the Q-value is 0.8764, indicating that the visualization map has a vital effect of dividing clusters. The top 12 highly cited papers are sorted by the number of citations, as shown in **Table 2**. The top 10 co-citation references were shown in **Supplementary Table 7**.

**Table 2:** Top 12 highly cited papers in ^18^F-FDG PET/CT radiomics research from WoSCC.

Rank	First Authors	Article Titles	Source	Hot Paper Status	Times Cited	Document Type
1	Zwanenburg A	The Image Biomarker Standardization Initiative: Standardized Quantitative Radiomics for High-Throughput Image-based Phenotyping	Radiology	Yes	613	Original Article
2	Yip SS	Applications and Limitations of Radiomics	Physics in medicine and biology	No	496	Review
3	Coroller TP	CT-Based Radiomic Signature Predicts Distant Metastasis in Lung Adenocarcinoma	Radiotherapy and oncology	No	427	Original Article
4	Nioche C	LIFEx: A Freeware for Radiomic Feature Calculation in Multimodality Imaging to Accelerate Advances in the Characterization of Tumor Heterogeneity	Cancer research	No	344	Original Article
5	Chicklore S	Quantifying Tumor Heterogeneity in ^18^F-FDG PET/CT Imaging by Texture Analysis	European journal of nuclear medicine and molecular imaging	No	313	Review
6	Leijenaar RT	Stability of FDG-PET Radiomics Features: An Integrated Analysis of Test-Retest and Inter-Observer Variability	Acta oncologica	No	279	Original Article
7	Vallieres M	Radiomics Strategies for Risk Assessment of Tumor Failure in Head-and-Neck Cancer	Scientific reports	No	191	Original Article
8	Larue RT	Quantitative Radiomics Studies for Tissue Characterization: A Review of Technology and Methodological Procedures	British journal of radiology	No	180	Review
9	Lee G	Radiomics and Its Emerging Role in Lung Cancer Research, Imaging Biomarkers and Clinical Management: State of the Art	European journal of radiology	No	161	Original Article
10	Orlhac F	A Postreconstruction Harmonization Method for Multicenter Radiomic Studies in PET	Journal of nuclear medicine	No	142	Original Article
11	Lucia F	Prediction of Outcome Using Pretreatment ^18^F-FDG PET/CT and MRI Radiomics in Locally Advanced Cervical Cancer Treated With Chemoradiotherapy	European journal of nuclear medicine and molecular imaging	No	117	Original Article
12	Valdora F	Rapid Review: Radiomics and Breast Cancer	Breast cancer research and treatment	No	112	Review

### Citation burst analysis

**Figure 9:**
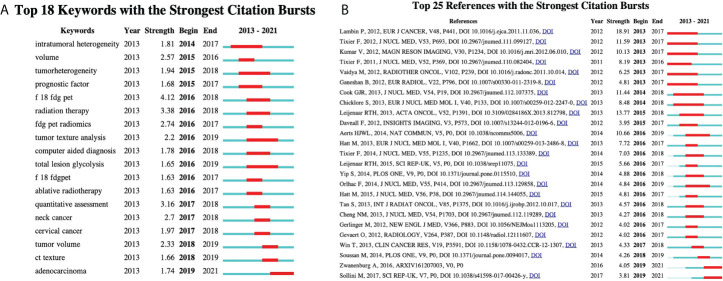
Detection of citation bursts. **(A)** Top 18 keywords with the strongest citation bursts. **(B)** Top 25 reference with the strongest citation bursts. *Begin* and *end* indicate the start time and end time of the mutation, respectively, and *strength* is the keyword mutation strength. The higher the strength, the greater the influence.

Keywords with the strong citation burst can show the transfer of research frontiers in different periods and judge potential development trends and frontier research. As shown in **Figure 9**, *begin* and *end* indicate the start time and end time of the mutation, respectively, and *strength* is the keyword mutation strength. The higher the strength, the greater the influence. The keyword citation burst map contains the 18 most essential keywords in ^18^F-FDG PET/CT radiomics field. The keyword with the highest strength is “F 18 FDG PET” (strength = 4.12), pointing out the theme of research. The most crucial keyword might be “radiation therapy” and “quantitative assessment” with the intensity of 3.38 and 3.16, respectively. They started to become important from 2016 to 2018. The extended duration of each keyword is no more than 3 years, suggesting that new hot topics burst rapidly, while research on a specific topic is not very persistent. Exploring the timeline view, from 2013 to 2016, the main focus was on the methodologies of ^18^F-FDG PET/CT radiomics in the identification of tumor heterogeneity and tumor volume measurement; many studies focus on the determination of the efficacy of radiotherapy by ^18^F-FDG PET/CT radiomics in 2016. During this period, computer-aided tools gradually became one of the research hotspots. Since 2017, many studies on a specific type of tumor have begun to emerge. On a constant basis, the ^18^F-FDG PET/CT radiomics research of head and neck tumors, gynecological tumors, and adenocarcinoma has continued growth. Observing the citation burst of references, we find that Lambin et al. (2012) represented that literature has the highest intensity value of 18.91. The articles by Zwanenburg A (2016) and Sollini M (2017) have seen a surge in citations starting in 2019.

## Discussion

PET/CT radiomics can be defined as the transformation of PET/CT images into massive quantitative imaging features with the help of computer software and quantitative analysis using statistical and/or machine learning methods to screen out the most valuable radiomics features, so as to serve the clinic. At present, a large number of studies have focused on the application and value of various texture features in PET images, and satisfactory results have been achieved for tumors such as lymphoma, lung cancer, liver cancer, breast cancer, nasopharyngeal cancer, and cervical cancer (18, 25, 26). When the number of patients is insufficient, some studies use technical means, such as external cohort verification or even deep learning technology to obtain constructive results (27, 28). In addition, the PET radiomics signature model also showed good performance in gene prediction, assessment of tumor patient prognosis, and risk stratification (29, 30).

### General data

In recent years, ^18^F-FDG PET/CT radiomics research is getting active, and the temporal trends show continuing growth in the number of publications and citations, with the number reaching 146 and 302 in 2021. The reason may be that the application of PET/CT in a tumor is more and more affirmed by clinicians. As a prominent media for academic communication, 134 journals have published research on ^18^F-FDG PET/CT radiomics. *European Journal of Nuclear Medicine and Molecular Imaging* (IF9.236), *Frontiers in Oncology* (IF6.244), and *Cancers* (IF6.639) were the most productive ones, and *Journal of Nuclear Medicine* (IF10.057) had the highest IF. We found that most of the productive journals were classified as Q1 or Q2, which suggests that the aforementioned journals published high-quality research with convincing and mature results.

As the leading force, the United States is the most prolific country with the highest centrality. We identified the United States, China, Germany, Netherlands as double-high nodes of the visualization map, which means that the authors of these countries have completed high-level research. According to the cooperation relationship, scholars from the United States, Japan, and South Korea have close cooperation in ^18^F-FDG PET/CT radiomics, and, at the same time, American researchers also maintain close ties with researchers from European countries. Brazilian scholars, from South America, often work with scholars from Western Europe and East Asia. Scientists from China, Australia, and European countries collaborate the most. It is worth noting that the research in the two major countries in North America has a cooperation relationship with the research in the Middle East country (Iran). Another interesting point is that, even with the countries’ large volume of publications and despite some countries being geographically adjacent, collaboration in East Asia was still rare and limited. The research capabilities of most developing countries in the field of ^18^F-FDG PET/CT radiomics should be improved. Throughout the visualization map, research was primarily done by authors from European countries, North American countries, and East Asian countries. In South America, only Brazilian scientists are now paying attention to the ^18^F-FDG PET/CT radiomics-related topics; in the Middle Eastern and African countries, only Iran was included in our analysis based on WoSCC status.

For the research institutions, Memorial Sloan-Kettering Cancer Center and Southern Medical University, Maastricht University, H. Lee Moffitt Cancer Center and Research Institute, and Humanitas University have led numerous high-quality studies. Among the top 5 institutions, the Memorial Sloan-Kettering Cancer Center was the foremost, with a frequency of 120. Among the high-impact institutions, University of Groningen and Leiden University were from Netherlands. One possible reason might be attributed to the well-developed medical device industry in Netherlands (31). Companies, such as Royal Dutch Philips Electronics Ltd., have long maintained a global leading edge in the field of medical imaging, allowing Dutch scientists to continuously access cutting-edge technologies. As is acknowledged, the concept of radiomics was just proposed by Dutch scientists Lambin et al. (32) from the Maastricht University Medical Center (Netherlands) in 2012. University Hospital (USA), the Memorial Sloan-Kettering Cancer Center (USA), and Southern Medical University (China) served as an outstanding bridge of communication in our visualization map, suggesting that multi-central innovative research in the future might be conducted under the cooperation of these institutions.

We found that nine authors published more than 10 papers, who have high academic reputations in ^18^F-FDG PET/CT radiomics research and analog research and have contributed significantly to developments and advances. Mathieu Hatt at the University of Brest in France was the most productive author, followed by Dimitris Visvikis, one of the group members of Mathieu, indicating that a net of collaborations centered on Mathieu and his team has been formed in this field. Mathieu was dedicated to the application of machine learning in PET/CT in oncology, and one of his studies was placed in the top 0.1% of papers in the academic field of Clinical Medicine by WoSCC (33). This hot paper standardized a set of 174 radiomic features, finding that 169 of the features were able to standardize widely, for example, mean, skewness, excess kurtosis, and a minimum of the intensity-based statistics family. Another study (34), cited 117 times, found that radiomics features of PET have higher prognostic power than usual clinical parameters and can be regarded as independent predictors of recurrence and loco-regional control of locally advanced cervical cancer.

### Research hotspots and Frontiers

The core content of radiomics is extracting features from quantitative imaging through AI-based high-throughput analysis and evaluating the metabolic biological behavior of particular tissues non-invasively. The comprehensive workflow of radiomics follows these steps: 1) acquisition and reconstruction of image, 2) establishment of data sets, 3) segmentation of image, 4) extraction of feature, 5) dimension reduction, 6) construction of a predicting model, and 7) validation of models using internal and/or external data sets. What is particular is that ^18^F-FDG PET/CT radiomics has a special attribute, distinct from other medical imaging modalities, e.g., CT radiomics, MRI radiomics, or radiomics in endoscopic examinations. Above all, when dealing with the quantitative imaging segment, PET functional imaging should be integrated with CT anatomical imaging. Then, during image acquisition, routine calibration of PET/CT equipment needs attention to avoid systematic errors caused by equipment status. We can clearly summarize the overall research contents of ^18^F-FDG PET/CT radiomics into two aspects. One is studying the technical know-how, i.e., accurate tumor segmentation and annotation, massive feature extraction and screening, and artificial intelligence model construction. The other is a discussion on typical clinical applications in oncology, i.e., intelligent diagnosis, efficacy evaluation, prognosis, and survival prediction. For example, Vallières et al. (35) used machine learning to develop a model to predict treatment effects based on ^18^F-FDG PET/CT images of 300 nasopharyngeal carcinoma patients and found that the developed model could predict local recurrence and distant metastasis with AUC of 0.69 and 0.86, respectively. Li et al. (36) analyzed the overall survival of 127 glioma patients using the Kaplan-Meier curve and log-rank test and concluded that there was a significant difference in predicting overall survival between high-risk and low-risk groups in their study.

Keywords that appear frequently can represent areas of focus in a given field (37), and the most cited papers are usually a concentrated expression of hot topics. Generally speaking, keywords with higher frequency of co-occurrence were radiomics, quantitative imaging, prediction, features, survival, and so on. One of the major concerns in ^18^F-FDG PET/CT radiomics is what extract features are used over time. There is no unified standard in extracting features of radiomics used in clinical practice. To match different research aims, scholars focus on various kinds of features (18). Commonly used ones include intensity feature, shape feature, texture feature, wavelet feature, clinical feature, etc. The intensity feature is a description of the concentration of PET/CT metabolic imaging agents, and the shape feature offers an insight into morphological change. These features are usually used in conjunction with textures (commonly including first-order gray histogram features and second-order high order). Almost all of the highly cited documents in the WOS database involve texture feature analysis. Among texture indicators, the gray-level co-occurrence matrix (GLCM) is the most commonly used feature calculation matrix, followed by the gray-level run-length matrix (GLRLM) and gray-level zone matrix (GLSZM). Due to the large amount of data, one of the most important tasks before modeling is dimensionality reduction. Wavelet features enhance certain characteristics of the image based on its frequency domain information. Studies use the wavelet feature in pretreatment and dimensionality reduction steps to increase the number of data input. Lue et al. (38) pointed out that features from high-frequency wavelet components were useful for the prediction of response to therapy. In their prognostic stratification model, the high-intensity run emphasis of PET in GLRLM wavelets serves as an independent predictive factor for treatment response. Other dimensionality reduction methods mainly include principal components analysis (PCA), linear discriminant analysis (LDA), and Laplace feature mapping.

We analyzed the clustering maps, finding that the hot research disease in ^18^F-FDG PET/CT radiomics studies is a malignant tumor and mainly contains lung cancer (45 articles), lymphoma (29 articles), hepatocellular carcinoma (26 articles), esophageal cancer (22 articles), breast cancer (19 articles), etc. suggesting that ^18^F-FDG PET/CT radiomics is likely to be a better tool for oncology research. According to the primary site of the tumor, the application of PET/CT radiomics in different diseases was categorized as follows: (i) Head and Neck Oncology: Applications in the field of head and neck oncology focus on the precise diagnosis of recurrence of carcinoma by ^18^F-FDG PET radiomics. A trial (39), covering 76 patients with nasopharyngeal carcinoma after treatment, showed that the AUC of PET radiomics features selection method and device cross-validation was 0.867~0.892 for distinguishing tumor recurrence from inflammatory response, which was excellent better than traditional metabolic parameters (AUC = 0.817). Wang et al. (16) performed an individualized diagnosis of tumor recurrence from radiation necrosis in glioma patients using an integrated ^18^F-FDG PET radiomics–based model, proposing that the AI model demonstrated good discrimination (AUC = 0.988, 95% CI: 0.975~1.000), and predictors contained in the individualized diagnosis model included the radiomics signature, the mean tumor-background ratio (TBR) of ^18^F-FDG, the maximum TBR of ^11^C-MET PET, and patient age. (ii) Thoracic and Chest Oncology: The research on thoracic cancer started early in the field of lung cancer and the related technologies are becoming relatively mature. With clustering keywords, i.e., survival and tumor heterogeneity, our analysis indicates that ^18^F-FDG PET/CT radiomics is commonly used to improve the ability to distinguish benign and malignant lung lesions, and the technology is also helpful in the prognostic analysis of lung cancer patients. Radiomics features derived from ^18^F-FDG PET/CT were associated with local control in patients with non–small cell lung cancer (NSCLC) undergoing stereotactic body radiation therapy (SBRT), and radiomics data can be used as predictors of  overall survival (OS), disease-specific survival, and regional control (40). Quantitative imaging features of lung cancer, such as volume, density, and metabolic activity, have been employed to enhance interpretation and improve the prognostic value (41). As to tumor heterogeneity, the deep learning score of EGFR mutation provides a non-invasive method for identifying NSCLC patients sensitive to EGFR tyrosine kinase inhibitors or immune checkpoint inhibitors treatments (19). Another significant application of ^18^F-FDG PET/CT radiomics in thoracic oncology is in esophageal cancer studies. Radiomics-guided ^18^F-FDG PET/CT scans can accurately predict the response of neoadjuvant chemoradiotherapy in esophageal cancer patients (42). Combining the radiomic features and traditional parameters, ^18^F-FDG PET/CT radiomics may also enable better stratification of patients with esophageal squamous cell carcinoma treated with neoadjuvant chemoradiotherapy and surgery into subgroups with various survival rates (43). ^18^F-FDG PET/CT radiomics is helpful in pathological classification, differential diagnosis, and prognosis prediction of breast cancer. A significant correlation exists between imaging features and the histological type of breast cancer. These features include standard parameters such as mean standard uptake value (SUVmean), total glycolysis of lesions (TLG), metabolic tumor volume (MTV), and advanced imaging features (histogram-based and shape and size features). As for chest tumors, Cheng et al. (44) retrospectively analyzed the PET-based radiomics machine learning model to predict axillary lymph node status in early-stage breast cancer, finding that the PET-based robust machine learning model integrating the clinical characteristics can predict the pathological node status and improve the true positive and true negative rate of pathological classification. The key to differential diagnosis using PET/CT images is to examine changes in tissue metabolism and uptake, and radiomics can extract quantitative variables that cannot be visually assessed in medical images (45). A preliminary study (46) of differential diagnosis used machine learning to differentiate breast carcinoma from breast lymphoma, finding that models based on clinical, SUV, and radiomics features of ^18^F-FDG PET/CT images have promising discriminative abilities. Lymphadenopathy is commonly found after the injection of the COVID-19 mRNA vaccine. Based on K-nearest neighbors and random forest models, Eifer et al. (47) considered that ^18^F-FDG PET/CT radiomics may have a role in differentiating benign nodes from malignant ones. Studies also keep on trying to identify radiomic prognosis predictors from PET/CT in breast cancer patient therapeutic efficacy. As for some prognostic factors, e.g., human epidermal growth factor receptor 2 (HER2)–positive, P53 mutation, Ki-67 proliferation index, and ^18^F-FDG PET/CT radiomics, data have the predictive and prognostic ability for personalized management. PET parameters showed stronger correlations with immunohistochemical factors and immunohistochemical subtype of breast cancer. Texture analysis indicates that HER2-positive tumors had significantly higher uptake of FDG, whereas luminal B–like/HER2^+^ and HER2-positive non-luminal tumors also showed more regional heterogeneity than Luminal A–like tumors on breast PET image (48). In the situation of the presence of p53 mutation, the use of ^18^F-FDG PET/CT radiomics data is believed to contribute to breast cancer management (49). Ha et al. used unsupervised clustering to figure out imaging biomarkers for estimation of intratumoral heterogeneity in locally advanced breast cancer, proposing that metabolic radiomics patterns are associated with Ki-67 expression (50). (iii) Abdominopelvic Oncology: The highly cited papers of abdominal tumor studies focused on cervical cancer, verifying that the radiomics model has higher prognostic power than usual clinical parameters (51), and Lucia et al. (26) put forward that entropy GLCM and GLRLM from functional imaging PET might be independent predictors of recurrence and loco-regional control in cervical cancer patients. Liu et al. (52) developed a predictive model by including 351 patients with stages IB to IIA squamous cell carcinoma of the uterine cervix and found that squamous cell carcinoma antigen level and pelvic lymph node SUVmax were independent predictors of pelvic lymph node metastasis, and the resulting line graph showed high sensitivity (70.5%), specificity (94.4%), and positive predictive value (93.9%). Other research on abdominopelvic cavity demonstrates that ^18^F-FDG PET/CT texture analysis can effectively differentiate renal cell carcinoma from renal lymphoma, and differential response after the first-line treatment of colorectal cancer patients (53, 54). On the basis of citation bursts analysis, we list some recent research status of different clinical applications of ^18^F-FDG PET/CT radiomics studies below: (i). The most crucial keyword “^18^F-FDG PET” started to become important in 2016, indicating that medical workers have started to place emphasis on clinical applications of ^18^F-FDG PET/CT radiomics ever since 2016. (ii) Intelligent diagnosis (keyword: computer-aided diagnosis, burst: 2016~2018), response evaluation (keyword: quantitative assessment, burst: 2017~2018), and survival prediction for cancer prognosis (keyword: prognostic factor, burst: 2015~2017) are regarded as typical applications of radiomics. One of the most cited studies concerned about whether the composite textures from the combination of FDG-PET and MR imaging information could quantitatively identify aggressive tumors at diagnosis (55). It verified that assessing lung metastasis risk of soft-tissue sarcomas through ^18^F-FDG PET/CT radiomics could improve patient outcomes. In some respects, response assessment after treatment can provide a reliable basis for predicting expected survival. The most cited study (56) on efficacy assessment and survival prediction reported that baseline ^18^F-FDG PET scan uptake in NSCLC, showing abnormal texture as measured by coarseness, contrast, and busyness, is associated with nonresponse to chemoradiotherapy by response evaluation criteria in solid tumors and with poorer prognosis. (iii) The ^18^F-FDG PET/CT radiomics analysis in adenocarcinoma can be speculated to become an emerging academic trend in clinical practice. During the last 3 years, the keyword “adenocarcinoma” was the most concerned and frequently cited. Adenocarcinoma research involves a primary focus on identification and image segmentation, identification of heterogeneity, prediction of recurrence and metastasis, evaluation of treatment efficacy, and prediction of survival (57–59). In clinical practice, the research puts out that the accuracy of the PET/CT imaging analyzed by radiomics in pathological pattern and staging is close to or even equal to the “gold standard” of pathological biopsy (60). We can speculate that ^18^F-FDG PET/CT radiomic is providing promising performance to move the post-operative pathological characterization analysis of adenocarcinoma forward to pre-operation.

In the co-citation map, it is worth mentioning that many scholars have a co-citation relationship in studies related to imaging biomarkers, texture analysis, and immunotherapy simultaneously. Texture analysis has long been used in the classification of correlations between imaging parameters of glucose metabolism and the expression levels of genomic biomarkers from cancers (55, 61). Furthermore, there are pros and cons in a variety of ^18^F-FDG PET/CT radiomic research. By convention, traditional machine learning methods, represented by the random forest, decision tree, and regression algorithms, are most commonly used to segment images. Such kind of manual segmentation is easy to operate, and we can obtain intuitive images for radiomics analysis. However, Ding et al. (37) pointed out that these algorithms mostly require a huge amount of matrix manipulations. It takes too much time and the results are unavailable to repeat. To note, the landmark literature of our citation burst especially put emphasis on the reproducibility of FDG-PET radiomics features, which indicates that it is of great necessity to control the variability between test-retest and inter-observer to make sure that all features are robustly measured. Learning-based algorithms are developed to solve this problem. Tixier et al. (62) studied the reproducibility of tumor uptake heterogeneity characterization through the fuzzy locally adaptive Bayesian (FLAB) algorithm; meanwhile, they used parameters on local and regional scales to replace simple SUV measurements, attaining similar or better reproducibility than simple SUV measurements. Learning-based algorithms have made substantive progress. Notwithstanding, it is important to pay attention that the results of these algorithms remain to be interpreted carefully, for most of the machine learning evaluations included insufficient numbers of patients and few studies have external validation on those developed AI models. Accessibility of data and software should be improved for researchers to expand the sample to verify the previous research results (27).

### Strengths and limitations

Although there has been a surge in bibliometric studies, knowledge visualization maps of PET/CT imaging are still very rare. We often find limitations such as unprofessional literature retrieval, lack of quality control in map interpretation, and incomplete reporting of original data, which affected the credibility of literature results and conclusions. Note that this study carried out quality control through interviews with librarians, and we listened to the literature retrieval strategies and suggestions provided by professionals. During the literature retrieval process, we included literature as comprehensive and complete as possible. When interpreting the co-occurrence analysis results, we invited three nuclear medicine physicians to offer suggestions to improve the reliability of the study. After a series of quality control procedures, it is reasonable to believe that the results of this study truthfully reflect the current research hotspots and frontiers of ^18^F-FDG PET/CT radiomics, and the research conclusions are true and reliable. This study is the first comprehensive literature analysis focusing on PET/CT radiomics research with a specific imaging technique. We hope that this study can provide a practical reference for scholars to use ^18^F-FDG imaging tracer to carry out PET/CT radiomics practice.

At the same time, we also recognize that there are some shortcomings and deficiencies in this research. For example, WoSCC was the only database to collect literature, and PubMed, Embase, CNKI, or other databases would be further needed in a comprehensive analysis. It is worth noting that WoSCC is the most commonly used database in bibliometrics research. Due to the limited number of documents available from the WoSCC, our study is likely not to contain an exhaustive number of records. Additionally, our research is conducted on an annual basis, so studies published in the first half of 2022 were not included, which may lead to missing some real-time hot issues.

## Conclusions

In conclusion, the current research status revealed that ^18^F-FDG PET/CT radiomics research has great development potential, with an increasing number publications and citations. So far, it has received significant attention in intelligent diagnosis, response evaluation, and survival prediction for cancer prognosis. *European Journal of Nuclear Medicine and Molecular Imaging*, *Frontiers in Oncology*, and *Cancers* were considered the top journals. United States, China, Germany, and Netherlands contribute a lot and have played vital roles in developing and expanding this field. The hot research topics in ^18^F-FDG PET/CT radiomics studies are malignant tumors. It is also necessary to focus on ensuring the reproducibility of ^18^F-FDG-PET radiomics features. Adenocarcinoma was the most concerned and frequently cited research direction. It is expected to become the frontier and development trend in the future.

For the first time, this study comprehensively screened literature associated with ^18^F-FDG PET/CT radiomics and provided a theoretical basis for the field of it, which may benefit researchers to deeply understand the current status of the field and encourage them to engage in the hotspots and frontiers. ^18^F-FDG PET/CT radiomics provides clinicians with a more accurate evaluation of treatment outcomes and more meaningful prognostic risk scores. It is foreseeable that using ^18^F-FDG PET/CT radiomics is expected to rapidly advance the development of molecular imaging on the basis of existing mature technology and further integrate nuclear medicine findings into clinical diagnosis and treatment aid decision-making.

## Data availability statement

The original contributions presented in the study are included in the article/**Supplementary Material**. Further inquiries can be directed to the corresponding authors.

## Author contributions

XL conceived and designed the structure of this manuscript. XL, XH, XY, PL, CG, and YW wrote the paper. JC, PW, and DL revised the paper. All authors contributed to the article and approved the submitted version.

## Funding

This study was funded by the National Natural Science Foundation of the People’s Republic of China, NSFC (grant number: 81571712), Zunyi Medical College Research Start Fund 2018ZYFY03, and QianKeHe platform talents [2017] (grant number: 5733-035).

## Acknowledgments

The authors would like to express their appreciation to Prof. Chaomei Chen, who invented CiteSpace and made it open for use. We’d also like to thanks Lecturer Jimei Cai (the information department of Zunyi Medical University Library, Guizhou Province, China) for her kindly receiving our interview on the strategy of data searching.

## Conflict of interest

The authors declare that the research was conducted in the absence of any commercial or financial relationships that could be construed as a potential conflict of interest.

## Publisher’s note

All claims expressed in this article are solely those of the authors and do not necessarily represent those of their affiliated organizations, or those of the publisher, the editors and the reviewers. Any product that may be evaluated in this article, or claim that may be made by its manufacturer, is not guaranteed or endorsed by the publisher.
