# Supplementary material for: Frontiers and hotspots of 18F-FDG PET/CT radiomics: A bibliometric analysis of the published literature
Source: Front Oncol. 2022 Sep 13;12:965773. doi: 10.3389/fonc.2022.965773 (PMC9513237; doi:10.3389/fonc.2022.965773)
Supplement: Supplementary file 1 [file DataSheet_1.doc]

**Supplementary Material**

**Supplementary information 1.** List of Study protocol

**Study title:** Frontiers and Hotspots of 18F-FDG PET or PET/CT Radiomics: A Bibliometric Analysis of the Published Literature

**Authors:** Xinghai Liu, Xianwen Hu, Xiao Yu, Pujiao Li, Cheng Gu, Guosheng Liu, Jiong Cai, Pan Wang and Dandan L

1. Concepts

a. Bibliometrics: bibliometric is a science that uses quantitative methods such as mathematics and statistics to study the distribution laws and quantitative relationships of documents, which can not only help scholars comprehend the development trend of specific research fields, but also assess the contributions of journals, institutions, and countries in specific research fields.

b. CiteSpace: CiteSpace is a visualization software for measuring the literature in a specific field based on the co-citation analysis theory and the pathfinder algorithm in Java. It is designed as a tool for progressive knowledge domain visualization (Chen, 2004). It can show the evolution of knowledge structure and themes trends in systematic mapping, and scholars can identify the research frontiers characterized by collaborative (influence) network.

c. Frequency: frequency refers to the number of occurrences of the retrieved target word in the intellectual structure.

d. Centrality (Betweenness centrality): it refers to the ability to act as a mediator in the entire relationship network. High centrality is often considered as a turning point or pivotal point in a field.

e. Degree: it is the most direct measure to describe the centrality of nodes in network analysis. The larger the degree is, the higher the centrality of the node is, and the node is more important in the network.

f. Log likelihoodrate (LLR algorithm): log likelihood rate (one of the clustering tag word extraction algorithms). In short, the larger the LLR, the more representative the word is for this clustering.

g. N & E: In the visualized maps, N represents the number of visualization map nodes, while E represents the number of connections.

1. Methods

a. CiteSpace (5.8.R3) was the mainly used software:

- (To fulfill the Primary purpose) we used this software to find out intellectual structure and emerging topics.

①Co-occurrence keywork networks (timeline view）

②Burst analysis

- (To meet secondary aims) we used this software to produce collaborative (influence) networks.

①Dual-visualization map of disciplinary

②Countries/Institutions/Authors co-citation networks

③Co-citation document networks

b. VOSviewer 1.6.16 and Scimago Graphica 1.0.18 were used to assist construction visualized maps.

VOSviewer: Co-occurrence keywords networks, Collaborative research relationships between countries.

Scimago Graphica: World map depicting the contribution of each country.

c. OriginPro 2021, Microsoft Office 2016 were applied to show the data in detail.

-Bar chart

-Spider char

-Bubble plot

-Sankey diagram

3.Parameters

General CiteSpace parameters used for analysis:

CiteSpace parameters were as follows:

- Nodes Labeled:1.0%

- Pruning: Panthfinder

- Time span (2013–2021) with one slice per year

- Links (strength: cosine, scope: within slices)

- Selection criteria (g-index scale factor K=25; LRF=3.0, L/N=10, LBY=5, e=1.0)

**Supplementary information 2.** Supplementary Figures


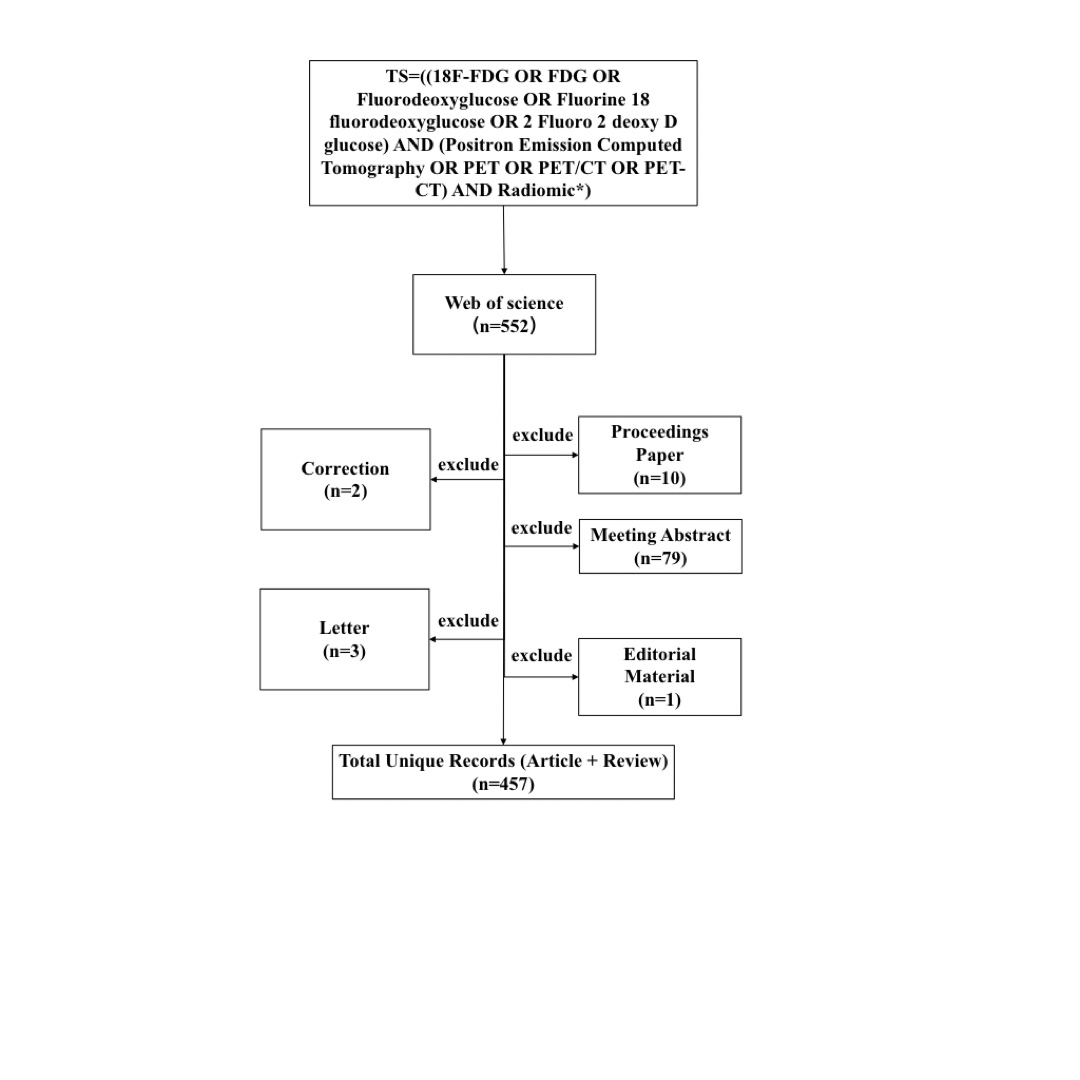


**Supplementary Figure 1.** Data searching strategy.

**
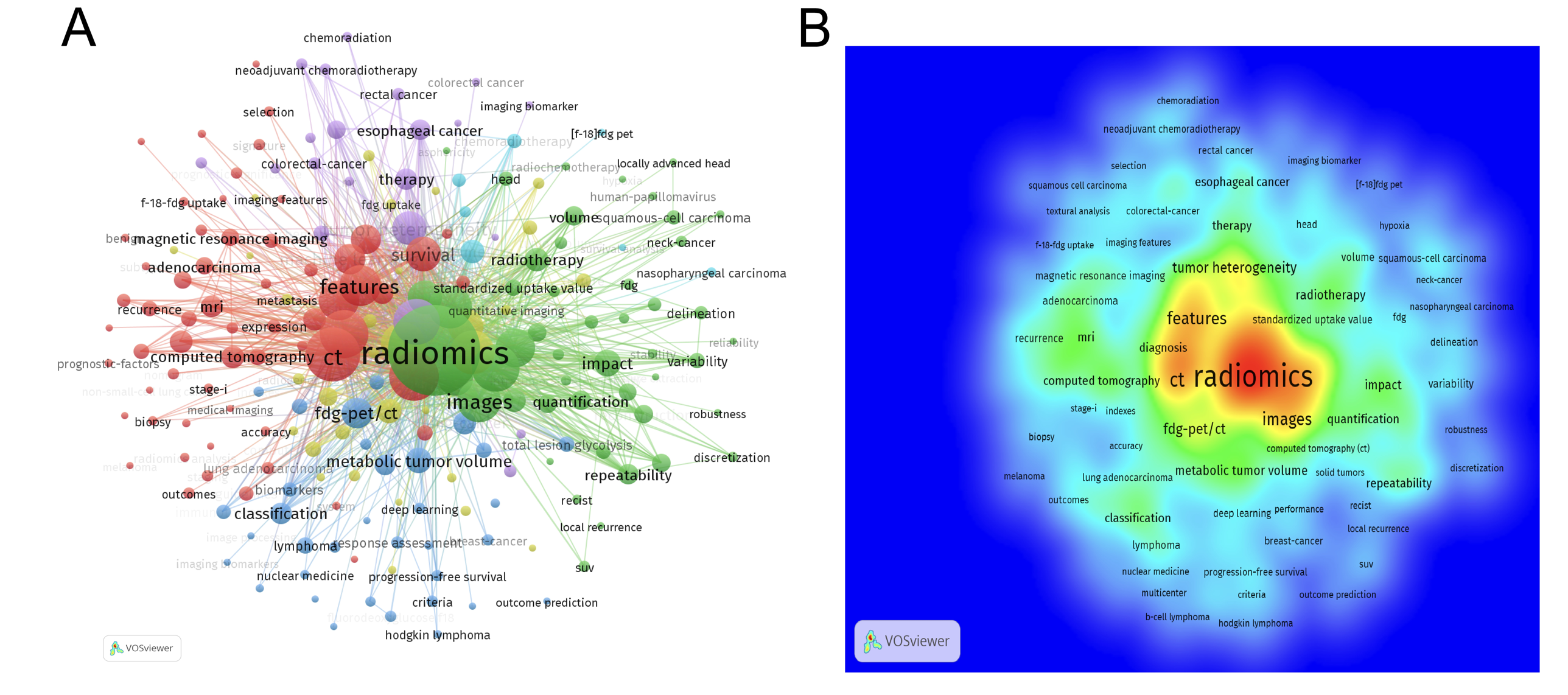
**

**Supplementary Figure 2.** Raw data of all keywords. (A) Default view of co-occurring keywords. (B) The density view of co-occurring keywords. The closer to red the keyword node color is, the higher the frequency of co-occurrence it has.

**Supplementary information 3.** Supplementary Tables

**Supplementary Table 1.** Document Type of ^18^F-FDG PET/CT radiomics research

| **No.** | **Document Type** | **value** |
| --- | --- | --- |
| 1 | Article | 361 |
| 2 | Correction | 2 |
| 3 | Editorial Material | 1 |
| 4 | Letter | 3 |
| 5 | Meeting Abstract | 79 |
| 6 | Proceedings Paper | 10 |
| 7 | Review | 96 |

*Notes: General Data from WoSCC (Total records found: 552 literatures; Total unique records (Oringinal Article + Review): 457; Duplicates: 0; Discarded: 95).*

**Supplementary Table 2.** The annual publications of ^18^F-FDG PET/CT radiomics research

| **No.** | **Years** | **Number** |
| --- | --- | --- |
| 1 | 2013 | 2 |
| 2 | 2014 | 1 |
| 3 | 2015 | 2 |
| 4 | 2016 | 21 |
| 5 | 2017 | 36 |
| 6 | 2018 | 62 |
| 7 | 2019 | 86 |
| 8 | 2020 | 101 |
| 9 | 2021 | 146 |

*Note: The search and download process was carried out on March 1, 2022, 9 years in total.*

**Supplementary Table 3. Full names of the institution shown in Fig.5**

| **Rank** | **Short name** | **Full name** |
| --- | --- | --- |
| 1 | Mem Sloan Kettering Canc Ctr | Memorial Sloan Kettering Cancer Center |
| 2 | Southern Med Univ | Southern Medical University |
| 3 | Maastricht Univ | Maastricht University |
| 4 | H Lee Moffitt Canc Ctr & Res Inst | The H. Lee Moffitt Cancer Center and Research Institute |
| 5 | Humanitas Univ | Humanitas University |
| 6 | Univ Groningen | University of Groningen |
| 7 | China Med Univ | China Medical University |
| 8 | Leiden Univ | Leiden University |
| 9 | Johns Hopkins Univ | Johns Hopkins University |
| 10 | German Canc Res Ctr | Deutsches Krebsforschungszentrum |
| 11 | Univ Hosp | University Hospital Brussels |
| 12 | Columbia Univ | Columbia University in the City of New York |
| 13 | Sichuan Univ | Sichuan University |
| 14 | Stanford Univ | Stanford University |
| 15 | Univ Brest | University of Brest |
| 16 | Wenzhou Med Univ | Wenzhou Medical University |
| 17 | Chinese Acad Sci | Chinese Academy of Sciences |
| 18 | Radboud Univ Nijmegen | Radboud University Nijmegen |
| 19 | Washington Univ | University of Washington |
| 20 | Fudan Univ | Fudan University |
| 21 | Univ Twente | University of Twente |

**Supplementary Table 4. Top 10 prolific authors**

| **No.** | **Frequency** | **Centrality** | **Authors** |
| --- | --- | --- | --- |
| 1 | 16 | 0.12 | MATHIEU HATT |
| 2 | 11 | 0 | DIMITRIS VISVIKIS |
| 3 | 9 | 0.01 | LIJUN LU |
| 4 | 9 | 0.08 | ARMAN RAHMIM |
| 5 | 8 | 0 | MARTINA SOLLINI |
| 6 | 8 | 0.05 | PHILIPPE LAMBIN |
| 7 | 7 | 0.1 | ESTHER G C TROOST |
| 8 | 7 | 0.03 | RONALD BOELLAARD |
| 9 | 6 | 0 | MARGARITA KIRIENKO |
| 10 | 6 | 0 | ARTURO CHITI |
| 11 | 6 | 0.05 | ROBERT J GILLIES |
| 12 | 5 | 0.02 | IRENE BUVAT |

**Supplementary Table 5.** **Top 10 cited authors**

| **No.** | **Frequency** | **Centrality** | **Authors** |
| --- | --- | --- | --- |
| 1 | 132 | 0.02 | Gillies RJ |
| 2 | 87 | 0 | Aerts HJWL |
| 3 | 80 | 0.02 | Hatt M |
| 4 | 72 | 0.02 | Leijenaar RTH |
| 5 | 72 | 0 | Hatt M |
| 6 | 65 | 0.02 | Lambin P |
| 7 | 56 | 0.02 | van Velden FHP |
| 8 | 55 | 0 | van Griethuysen JJM |
| 9 | 53 | 0.05 | Vallieres M |
| 10 | 52 | 0 | Nioche C |
| 11 | 46 | 0 | Leijenaar RTH |
| 12 | 46 | 0.02 | Chalkidou A |

**Supplementary Table 6.** The actual frequency numbers of clusters in Fig.7

| **ClusterID** | **Label(LLR)** | **Number** | **Proportion** |
| --- | --- | --- | --- |
| 0 | #0 f-18-fdg pet/ct | 36 | 12.00% |
| 1 | #1 breast cancer | 32 | 10.70% |
| 2 | #2 reconstruction | 27 | 9.60% |
| 3 | #3 standardized uptake value | 26 | 8.60% |
| 4 | #4 artificial intelligence | 22 | 7.30% |
| 5 | #5 hepatocellular carcinoma | 21 | 7.00% |
| 6 | #6 computed tomography | 19 | 6.30% |
| 7 | #7 risk stratification | 17 | 5.60% |
| 8 | #8 lung adenocarcinoma | 16 | 5.30% |
| 9 | #9 cervical cancer | 16 | 5.30% |
| 10 | #10 prostate cancer | 15 | 5.00% |
| 11 | #11 image analysis | 15 | 5.00% |
| 12 | #12 lung cancer | 10 | 3.30% |
| 13 | #13 somatostatin analog | 9 | 3.00% |
| 14 | #14 radiation oncology | 9 | 3.00% |
| 15 | #15 image | 8 | 2.60% |

**Supplementary Table 7.** Top 10 co-citation references in ^18^FDG PET-CT Radiomics research

| Cluster | First Author | Title | Source | Year | Frequency of citation |
| --- | --- | --- | --- | --- | --- |
| #11 | Gillies RJ | Radiomics: Images Are More than Pictures, They Are Data | Radiology | 2016 | 132 |
| #1 | Aerts HJ | Decoding tumour phenotype by noninvasive imaging using a quantitative radiomics approach | Nat Commun | 2014 | 87 |
| #7 | Hatt M | ^18^F-FDG PET Uptake Characterization Through Texture Analysis: Investigating the Complementary Nature of Heterogeneity and Functional Tumor Volume in a Multi–Cancer Site Patient Cohort | J Nucl Med | 2015 | 80 |
| #1 | Leijenaar RT | The effect of SUV discretization in quantitative FDG-PET Radiomics: the need for standardized methodology in tumor texture analysis | Sci Rep-UK | 2015 | 72 |
| #7 | Hatt M | Characterization of PET/CT images using texture analysis: the past, the present… any future? | Eur J Nucl Med Mol I | 2017 | 72 |
| #11 | Lambin P | Radiomics: the bridge between medical imaging and personalized medicine | Nat Rev Clin Oncol | 2017 | 65 |
| #0 | van Velden FH | Repeatability of Radiomic Features in Non-Small-Cell Lung Cancer [18F]FDG-PET/CT Studies: Impact of Reconstruction and Delineation | Mol Imaging Biol | 2016 | 56 |
| #11 | van Griethuysen JJM | Computational Radiomics System to Decode the Radiographic Phenotype | Cancer Res | 2017 | 55 |
| #15 | Vallieres M | A radiomics model from joint FDG-PET and MRI texture features for the prediction of lung metastases in soft-tissue sarcomas of the extremities | Phys Med Biol | 2015 | 53 |
| #5 | Nioche C | LIFEx: A Freeware for Radiomic Feature Calculation in Multimodality Imaging to Accelerate Advances in the Characterization of Tumor Heterogeneity | Cancer Res | 2018 | 52 |
